# Light-dependent expression of four cryptic archaeal circadian gene homologs

**DOI:** 10.3389/fmicb.2014.00079

**Published:** 2014-03-04

**Authors:** Michael Maniscalco, Jennifer Nannen, Valerie Sodi, Gillian Silver, Phillip L. Lowrey, Kelly A. Bidle

**Affiliations:** Department of Biology, Rider UniversityLawrenceville, NJ, USA

**Keywords:** archaea, halophiles, gene expression, mutants

## Abstract

Circadian rhythms are important biological signals that have been found in almost all major groups of life from bacteria to man, yet it remains unclear if any members of the second major prokaryotic domain of life, the Archaea, also possess a biological clock. As an initial investigation of this question, we examined the regulation of four cyanobacterial-like circadian gene homologs present in the genome of the haloarchaeon *Haloferax volcanii*. These genes, designated *cirA, cirB, cirC, and cirD*, display similarity to the KaiC-family of cyanobacterial clock proteins, which act to regulate rhythmic gene expression and to control the timing of cell division. Quantitative RT-PCR analysis was used to examine the expression of each of the four *cir* genes in response to 12 h light/12 h dark cycles (LD 12:12) in *H. volcanii* during balanced growth. Our data reveal that there is an approximately two to sixteen-fold increase in *cir* gene expression when cells are shifted from light to constant darkness, and this pattern of gene expression oscillates with the light conditions in a rhythmic manner. Targeted single- and double-gene knockouts in the *H. volcanii cir* genes result in disruption of light-dependent, rhythmic gene expression, although it does not lead to any significant effect on growth under these conditions. Restoration of light-dependent, rhythmic gene expression was demonstrated by introducing, *in trans*, a wild-type copy of individual *cir* genes into knockout strains. These results are noteworthy as this is the first attempt to characterize the transcriptional expression and regulation of the ubiquitous *kaiC* homologs found among archaeal genomes.

## Introduction

Life on Earth is challenged by 24 h environmental oscillations, the most prevalent of which are the light/dark cycle and temperature fluctuations. To anticipate and respond appropriately to these recurrent environmental stimuli, organisms have evolved endogenous, cell-autonomous, self-sustained circadian clocks. These biological timekeepers drive circadian rhythms in biochemistry, gene expression, physiology and behavior and synchronize them to environmental time cues. The strong selective pressure for the precise temporal coordination of internal biological processes with the external day is evident in the diverse phylogenetic distribution of circadian clocks-from unicellular cyanobacteria, to fungi, plants, insects, and vertebrates, including humans (Dunlap et al., 2004).

To date, the only prokaryotes shown definitively to possess a circadian clock are the cyanobacteria (Johnson, 2007; Dong and Golden, 2008). This clock regulates many processes in the cyanobacterial cell, including global gene expression and cell division (Liu et al., 1995; Mori et al., 1996), and enhances reproductive fitness (Ouyang et al., 1998; Woelfle et al., 2004). The central clock of the cyanobacterium *Synechococcus elongatus* PCC 7942 is composed of just three proteins: KaiA, KaiB, and KaiC. Inactivation of any *kai* gene results in arrythmicity (Ishiura et al., 1998). It was initially proposed that, similar to the general model emerging in eukaryotes, the circadian mechanism in cyanobacteria was based on rhythmic *kaiBC* transcription and translation (Nakahira et al., 2004). The dramatic demonstration, however, that the self-sustained, temperature-compensated rhythm of KaiC phosphorylation could be reconstituted *in vitro* by mixing ATP with all three Kai proteins, strongly suggested that the primary pacemaker in the *Synechococcus* clock system is a posttranslational phosphorylation cycle rather than a transcriptional/translational feedback loop (Nakajima et al., 2005). Recent results, however, indicate that both a transcriptional/translational feedback loop and posttranslational phosphorylation cycle are necessary to maintain precise and robust circadian rhythms in cyanobacteria (Kitayama et al., 2008).

Analyses of sequenced archaeal genomes reveal an abundance of putative homologs of the cyanobacterial *kaiC* gene (Leipe et al., 2000; Dvornyk et al., 2003; Dvornyk and Knudsen, 2005; Ming et al., 2007) although no clear homologs of *kaiA* or *kaiB* are found among Archaea. Indeed, the crystal structure of a KaiC homolog from the hyperthermophilic achaeon, *Pyrococcus horikoshii* OT3, has been determined (Ming et al., 2007; Kang et al., 2009), yet no functional analyses have been reported to date. Given that many members of the Archaea and Bacteria inhabit overlapping ecological niches, and given the pervasiveness of horizontal gene transfer among prokaryotes (Garcia-Vallve et al., 2000; Gogarten et al., 2002; Koonin and Wolf, 2008), it is not surprising that some Archaea harbor cyanobacterial-like circadian genes. What has yet to be determined, however, is whether these putative *kai* homologs are expressed, and if so, how they function in the Archaea.

In this report, we describe the characterization of four cryptic *kaiC*-like genes found in the halophilic archaeon, *Haloferax volcanii*, to determine how these genes are expressed and potentially regulated in response to cycles of light and dark. Here, we demonstrate the expression of these genes and their oscillating, light-dependent regulation using quantitative RT-PCR in an examination of RNA isolated from *H. volcanii* cultures grown in light/dark cycles. Targeted gene knockouts in three of the four genes reveal a disruption of this diurnal, light-dependent regulation in remaining wild-type genes, indicating that each of these genes is functionally required for this light-driven, regulatory pattern. The implications for these results are important, as this is the first functional demonstration, to our knowledge, of light/dark-driven transcriptional regulation in *kaiC*-like genes in the third major domain of life, the Archaea.

## Materials and methods

### Strains, plasmids, and culture conditions

Strains and plasmids used for strain construction are found in Table 1. *Haloferax volcanii* strain DS70 (Wendoloski et al., 2001) was grown aerobically with shaking at 45°C in an incubator equipped with a programmable photosynthetic light bank (Innova 42R, Eppendorf). Cells were cultured in medium containing 125 g NaCl, 45 g MgCl_2_.6H_2_O, 10 g MgSO_4_.7H_2_O, 10 g KCl, 1.34 ml 10% CaCl_2_.2H_2_O, 3 g yeast extract, and 5 g tryptone, per liter (Robb et al., 1995). For constant light (LL) or constant dark (DD) conditions, liquid cultures were inoculated to a starting O.D._600_ of 0.1 from 48-h starter cultures, grown until mid-exponential phase, and then harvested by centrifugation for 5 min at 4°C, 6000 × g. For light and dark treatments (LD 12:12), cells were maintained in balanced growth conditions. To achieve balanced growth, an O.D._600_ reading was taken of an actively growing starter culture which was then diluted with fresh media to an O.D._600_ = 0.8 to initiate the experiments. This procedure was repeated every 12 h. Under these conditions, O.D._600_ values never varied more than 0.6–0.8 units from the end of one 12 h growth period before the addition of fresh media to the beginning of the next 12 h growth period after the addition of fresh media, ensuring cells remained in mid-exponential phase through the duration of the experiments. At 12 h time points, aliquots of cell cultures were centrifuged, supernatants were removed and pellets were stored at −80°C until processing for RNA. The amount of light was measured, in lux units, using a hand-held light meter (Reliability Direct, Inc., VWR). During dark growth conditions, lux units were equal to 0, while light growth conditions equaled 3000 lux.

**Table 1:** **Table of strains and plasmids used in this study**.

**Strains**	**Description**	**Source or reference**
DS70	Wild-type isolate DS2 cured of plasmid pHV2	Wendoloski et al., 2001
H26	DS70 Δ*pyrE2*	Allers et al., 2004
JN1	H26 (Δ*cirB*)	This study
JN2	H26 (Δ*cirC*)	This study
JN3	H26 (Δ*cirD*)	This study
GL1	JN2 (Δ*cirBcirC*)	This study
GL2	JN3 (Δ*cirBcirD*)	This study
GL3	JN3 (Δ*cirCcirD*)	This study
**PLASMIDS**		
pTA131	Amp^r^; pBluescript II containing P_*fdx-pyrE*2_	Allers et al., 2004
pTA354	*E. coli/H. volcanii* pTA131-based shuttle vector, with *pyrE2* marker and pHV1/4 replication origin	Norais et al., 2007
pMA7	pTA131with *cirB* fragment containing a 250 bp deletion within its coding region	This study
pMA8	pTA131with *cirC* fragment containing a 500 bp deletion within its coding region	This study
pAB6	pTA131with *cirD* fragment containing a 122 bp deletion within its coding region	This study
pMM2	pTA354 with ~1.4 kb full-length *cirB* gene and 5′ and 3′ flanking regions	This study
pMM3	pTA354 with ~1.4 kb full-length *cirC* gene and 5′ and 3′ flanking regions	This study
pMM4	pTA354 with ~1.4 kb full-length *cirD* gene and 5′ and 3′ flanking regions	This study

### Phylogenetic tree construction

Sequences used to construct a phylogenetic tree of cyanobacterial KaiC proteins and archaeal KaiC homologs were obtained by performing a protein BLAST search of microbial genomes within GenBank on the NCBI website. Sequence alignments were performed using CLUSTALW in MEGA6 (Tamura et al., 2013). The Maximum Likelihood method based on the JTT matrix-based model (Jones et al., 1992) was used to create a tree in MEGA6 with the highest log likelihood (−5876.7) in MEGA6. Initial tree(s) for the heuristic search were obtained by applying the Neighbor-Joining method to a matrix of pairwise distances estimated using a JTT model. The tree is drawn to scale, with branch lengths measured in the number of substitutions per site. The analysis involved 19 amino acid sequences and all positions containing gaps and missing data were eliminated. There were a total of 201 positions in the final dataset.

### RNA isolation and cDNA synthesis

RNA isolation and cDNA synthesis were performed as previously described (Bidle et al., 2008). Briefly, *H. volcanii* cells were harvested, resuspended in Tri Reagent (MRC, Cincinnati, OH) and processed according to the manufacturer's protocol. RNA was resuspended in RNAse-free water and treated with TURBO DNase (Ambion, Austin, TX). To ensure contaminating DNA was removed in the previous step, a 30-cycle PCR reaction was performed on purified RNA using the primer sets designed for qRT-PCR analyses, *cirA* (F, 5′-GCCTGTATCTCACCTTCGAAG; R, 5′-GTTCTTGATGCTCTGCTTGC), *cirB* (F, 5′-CGTCTACATCACGCTCGAAG; R, 5′-CCTCGTTCGTGAGTTCGTAC), *cirC* (F, 5′-CGAACCGAACGTACATGG; R, 5′-GAACTTCTCGATGCGGAC), or *cirD* (F, 5′-GGTTCGACGAGCTCATTC; R, 5′-GCTCACGAGGTTGATGAAG), as well as primers designed to amplify an ~400 bp region within the *H. volcanii* 16S rRNA gene (F, 5′-CGAAGGTTCATCGGGAAATCC; R, 5′- GTCATCACTGTAGTCGGAGC). DNA-free RNA samples were quantified and first-strand cDNA synthesis was initiated using 1 μ g total RNA primed with random hexamers from the AffinityScript QPCR cDNA synthesis kit according to the manufacturer's protocol (Stratagene, La Jolla, CA). Negative controls contained no reverse transcriptase in the first strand cDNA reaction.

### Quantitative RT-PCR

Transcript levels specific to each of the *cir* genes were analyzed using qRT-PCR for duplicate samples collected from replicate cultures of *H. volcanii* grown in several different conditions including LL, DD, or balanced growth in LD 12:12 cycles. Quantitative RT-PCR was performed using the RT SYBR Green qPCR Master Mix (SABiosciences, Fredrick, MD) and initiated by adding 1 μ l of the first-strand cDNA synthesis reaction to forward and reverse primers for either *cirA*, *cirB*, *cirC*, or *cirD*. Archaeal 16S rDNA amplification served as a normalizer. The reactions were performed using a RotorGene RG-3000 (Corbett Life Science, Qiagen) for 45 cycles at 95°C, 30 sec; 50°C, 1 min; 72°C, 1 min.

Standard curves for the reaction were created by amplifying each individual *cir* gene from genomic DNA and cloning into the pCR2.1-TOPO vector (Invitrogen). Serially diluted plasmid DNA served as a standard curve (avg. *r*^2^ value ~ 0.98) in subsequent qRT-PCR analyses. Amplification efficiency (efficiency = 10^(−1/slope)^) of all primer sets used was shown to be above 85%. *Cir* gene expression was normalized to 16 s rRNA expression and relative gene expression was calculated using the 2^−ΔΔCT^ method (Livak and Schmittgen, 2001).

### Creation of knockouts in *cir* genes

*H. volcanii* knockout strains were created using established gene disruption mutagenesis (“pop-in/pop-out” method) protocols that rely on transformant selection against a Δ *pyrE2* background (Bitan-Banin et al., 2003) using constructs made in pTA131, a pBluescript-based suicide plasmid containing the *pyrE2* gene as a selectable marker (Allers et al., 2004). These constructs contain ~200–300 bp of the 5′ region of the gene to be mutated fused to ~200–300 bp of the 3′ end of the gene, creating a deletion within the middle of each gene. To facilitate the creation of these constructs, PCR was used to amplify both the 5′ upstream and 3′ downstream regions of interest, with the resulting PCR products subsequently being cloned into pCR2.1-TOPO (Invitrogen, Carlsbad, CA). Positive clones were then digested with the enzyme *Eco*RI, gel purified, and ligated together. Ligation products were screened using the 5′ upstream gene's forward primer and 3′ downstream gene's reverse primer to amplify a sequence that contained the 5′ region concatenated to the 3′ region in the proper orientation (i.e., only correctly ligated products, those that run 5′ to 3′ with a truncation in the middle, will amplify). Following this, resulting PCR products were cloned into pCR2.1-TOPO, and positively identified clones were digested with the enzymes *Xho*I and *Bam*HI to facilitate cloning into the suicide vector pTA131. Simultaneously, each of the full-length *cir* genes, along with ~200 bp of flanking DNA on each 5′ and 3′ end, were cloned into the autonomously replicating shuttle vector pTA354 (Norais et al., 2007). These clones served both to monitor transformation efficiency during knockout strain construction, as well as to complement, *in trans*, the single gene knockouts in subsequent experiments. All constructs were subjected to DNA sequencing analysis prior to *H. volcanii* transformations to verify their DNA fidelity.

The *H. volcanii* uracil auxotrophic strain H26 [Δ *pyrE2;* (Allers et al., 2004)] was transformed with each construct using established methods (Cline et al., 1995). Positive transformants, displaying uracil prototrophy on selective medium, were subsequently patched onto Hv-CA plates (Hv-CA medium contains 0.5% (w/v) casamino acids, replacing the tryptone and yeast extract from the standard *H. volcanii* growth medium described above) containing 5-fluoroorotic acid (5-FOA) as uracil auxotrophs are unable to convert 5-FOA to its toxic analog, 5-fluorouracil. Thus, transformants that have lost the plasmid through homologous recombination events display 5-FOA resistance. Ura^−^ 5-FOA^R^ colonies were screened via PCR to determine if they harbored the wild-type or deleted version of each *cir* gene. The resulting knockout strains were confirmed via PCR and DNA sequencing analysis.

### Complementation of *cir* gene knockouts

Following knockout strain construction, full-length *cir* gene sequences were transformed into *H. volcanii* for complementation analyses. Each individual Δ *cir* knockout strain was transformed with the autonomously replicating shuttle vector harboring the wild-type gene (Table 1). Following successful transformation, clones were grown in the aforementioned LD 12:12 diurnal conditions and gene expression was examined via qRT-PCR as previously described.

## Results

### Identification of four *H. volcanii* circadian gene homologs

*H. volcanii* contains four cyanobacterial-like circadian rhythm gene homologs in its genome (Hartman et al., 2010), all displaying similarity to the KaiC-family of bacterial clock proteins. These genes were designated *cirA*, *cirB*, *cirC*, and *cirD* and they encode for predicted proteins of 27.5 kDa, 30.1 kDa, 52.7 kDa, and 29.7 kDa, respectively. These genes are not contained within an operon, but rather, are found distantly located from one another around the chromosome (Supplemental Figure 1). The *cirA* gene is located among a group of flagellar genes, while flanking the *cirB* gene are an NADH oxidase and ATP-NAD kinase; both *cirA* and *cirB* are divergently transcribed from their flanking genes. Interestingly, *cirC* is adjacent to *fix*L, an oxygen sensor protein; in cyanobacteria, *fixL* phosphorylates and dephosphorylates nitrogen fixation genes in response to environmental oxygen concentration (Monson et al., 1995). Directly upstream of *cirC* is the *orc2* gene that has been shown to participate in DNA replication initiation (Robinson and Bell, 2005), although a recent study questions the role of replication origins in archaea, as deletion analyses indicate they are not solely required for cell division (Hawkins et al., 2013). Flanking *cirD* are an oxido-reductase and a hypothetical protein.

**Figure 1:**
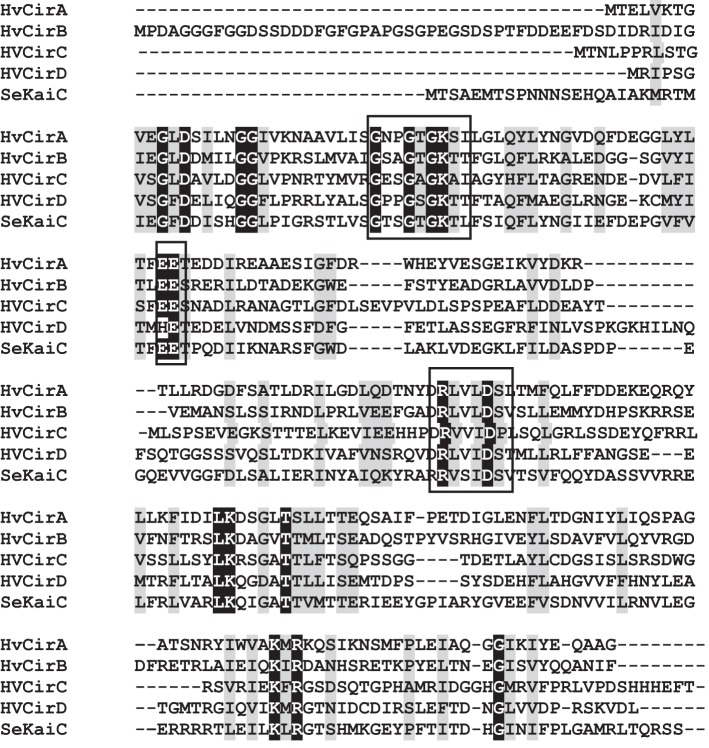
**Amino acid alignments of the N-terminal region of *S. elongatus* KaiC (SeKaiC) with *H. volcanii* (HV) *CirA*, *CirB*, *CirC*, and *CirD***. Predicted Walker A and B motifs, as well a conserved pair of catalytic glutamate residues,are boxed. Identical residues are shaded in black; similar residues are shaded in gray.

Each of these predicted protein sequences was aligned to *S. elongatus* KaiC, an ~58 kDa protein (Ishiura et al., 1998), and were shown to share between 28–33% identity and 48–55% similarity to this protein. More importantly, each of these predicted proteins contains a domain with a Walker A motif [(G/A)XXXXGK(T/S)] and Walker B [(RXXX(D/E)] motif where X = hydrophobic residue; (Walker et al., 1982), common to the KaiC family of proteins (Ishiura et al., 1998; Golden and Canales, 2003). These highly conserved regions are found within a diverse array of P-loop nucleoside triphosphate hydrolase protein families that, as well as KaiC, include RadA, RecA, and DnaB (Leipe et al., 2000). In addition to the Walker boxes found within *H. volcanii* Cir proteins, these predicted proteins also contained the conserved pair of catalytic glutamate residues hypothesized to be involved in ATP binding (Yoshida and Amano, 1995). The Walker A and B motifs and conserved glutamate residues are highlighted within the *H. volcanii* Cir alignments with *S. elongatus* KaiC (Figure 1). It should be noted that only *CirC* contains the double Walker A and Walker B domain structure found in *S. elongatus* KaiC; the remaining homologs, *CirA*, *CirB*, and *CirD* only posses a single Walker A and Walker B domain. When compared to each other, the predicted *H. volcanii* Cir proteins share between 25–30% identity and 44–54% similarity at the amino acid level (data not shown).

A more extensive search of other annotated archaeal genomes indicates that KaiC homologs are widespread among all three archaeal kingdoms (i.e., *Euryarchaeota, Crenarchaeota*, and *Nanoarchaeota*) in this domain of life, consistent with other reports (Leipe et al., 2000; Dvornyk et al., 2003; Dvornyk and Knudsen, 2005; Ming et al., 2007). Indeed, greater than half of the ~150 annotated archaeal genomes in the GenBank database have homologs to *S. elongatus* KaiC, most of which have a single Walker A and B domain structure (Dvornyk et al., 2003). Currently, their function remains unknown.

**Figure 2:**
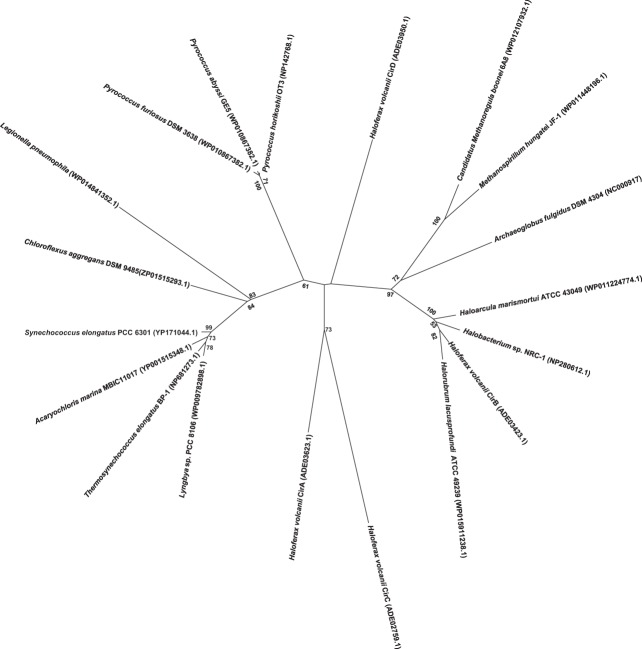
**Phylogenetic analysis of various known and predicted KaiC sequences from select cyanobacteria and archaea as compared with *H. volcanii* Cir sequences**. Sequence alignments and tree construction, using Maximum Likelihood, were performed using MEGA6 (Tamura et al., 2013). The tree is drawn to scale, with branch lengths measured in the number of substitutions per site. Accession numbers for the sequences used in the trees are provided in the figure.

In order to visualize the relationship among the four Cir protein sequences found in *H. volcanii*, a phylogenetic tree was constructed to include other select annotated archaeal KaiC homologs, representative cyanobacterial KaiC proteins, including *S. elongatus*, and a recently characterized KaiC homolog from *Legionella pneumophila*. Interestingly, investigations of the *L. pneumophila* KaiC homolog revealed that it does not possess circadian properties, but rather appears to play a role in stress adaptation in the organism (Loza-Correa et al., 2014). As shown in Figure 2, the cyanobacterial KaiC proteins cluster within one group, distinct from *Legionella* and *Chloroflexus*, a member of the green-non-sulfur bacteria and an anoxygenic phototroph. The *H. volcanii CirB* homologs falls among several other haloarchaeal genera, while *CirA*, and *CirC* group together and distinct from the other sequences in the tree. (Figure 2). Given the fact that these proteins are only 25–30% identical to each other, these results are unsurprising and suggest that if they did arise from a gene duplication event, it was not recent in the organism's history.

### Growth of *H. volcanii* in varying light conditions and establishment of light-based differential *cir* gene expression

Before initiating qRT-PCR experiments, it was first established that growth in constant light (LL) or dark (DD) conditions had no discernable effect on, or advantage for, the growth of wild-type *H. volcanii*. There is very little difference in growth rate or abundance between the two conditions, thus ruling out the possibility that any detectable increased *cir* gene expression could be attributed to growth rate (Supplemental Figure 2). We next wanted to determine if any of the *cir* genes were regulated in a light- or dark-dependent manner; for this, a quantitative RT-PCR approach was used. Transcript levels specific for each *H. volcanii cir* gene were analyzed in triplicate using qRT-PCR with RNA isolated from duplicate mid-exponential cultures grown until mid-exponential phase under DD or LL conditions. Results of these initial experiments demonstrated that transcript levels of the four *H. volcanii cir* genes were greater during growth in DD (ranging from ~2.1-fold in *cirB* to ~16.2-fold in *cirD*; data not shown), as compared with expression during LL conditions. These results demonstrate that the *H. volcanii cir* genes are regulated in a light-dependent manner.

**Figure 3:**
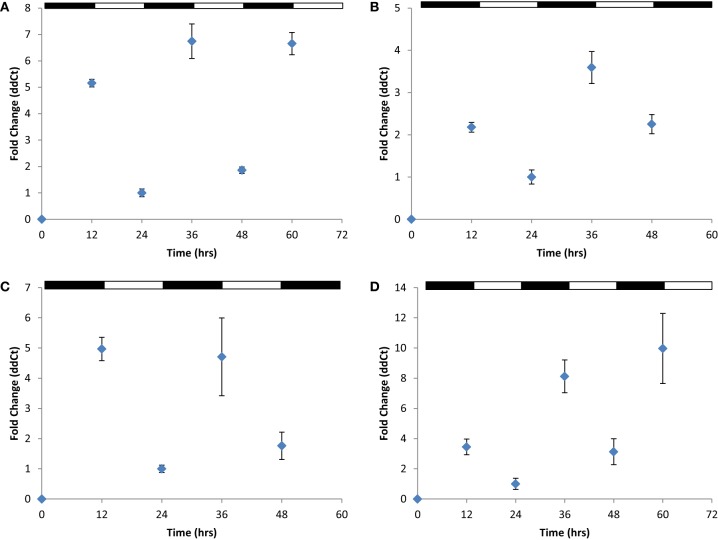
**Quantitative RT-PCR analysis of *cir* gene expression**. Expression of **(A)** *cirA*, **(B)** *cirB*, **(C)** *cirC*, or **(D)** *cirD* during synchronous growth in LD 12:12 cycles. Relative gene expression, as indicated by fold-change, was calculated using the 2^−ΔΔCT^ method (Livak and Schmittgen, 2001). Results given are pooled data collected from three independently-conducted experiments.

*H. volcanii* cells were next cultured in balanced growth conditions and grown under 12 h light/12 h dark conditions (LD 12:12; i.e., a typical diurnal cycle) for 72 h. These experiments demonstrated a consistent increase in gene expression for all four *cir* genes examined during each growth period in darkness (ranging from ~2.2-fold in *cirB* to ~10-fold in *cirD*) and decreased gene expression during growth in the light (Figures 3A–D). This pattern of regulation persisted regardless of whether the cycle was started in the light phase or the dark phase. To ensure that medium supplementation during the creation of balanced growth conditions was not altering the results of our studies, namely, affecting transcript abundance, we performed the following experiment. *H. volcanii* cells were cultured in constant LL or DD conditions and sampled every 3 h over a 12 h period. To maintain balanced growth, after every 3 h sampling period, cultures were supplemented with fresh growth medium to maintain the cells in mid-exponential phase. Results from these experiments revealed that gene expression remained constant over this sampling period (data not shown), indicating that the oscillations of gene expression seen in Figure 3 are not in response to environmental manipulations (i.e., changing growth medium every 12 h).

### How does a knockout in a *cir* gene affect rhythmic gene expression in the remaining wild-type *cir* genes?

**Figure 4:**
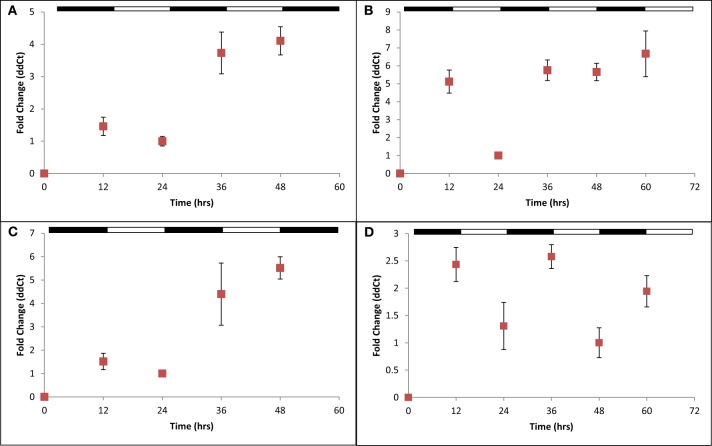
**Quantitative RT-PCR analysis of *cir* gene expression in select Δ*cir* mutants**. Expression of **(A)** *cirD* in JN1; **(B)** *cirB* in JN2; **(C)** *cirC* in JN3; and **(D)** *cirB* in JN1 (pMM2) during synchronous growth in LD 12:12 cycles. Loss of any single *cir* gene results in arrhythmic gene expression. Complementation of JN1 with an *in trans* copy of wild-type *cirB* (pMM2) restores rhythmic gene expression. Relative gene expression, as indicated by fold-change, was calculated using the 2^−ΔΔCT^ method (Livak and Schmittgen, 2001). Results given are pooled data collected from two independently-conducted experiments.

To address this question, we created knockout strains using established gene disruption protocols (Bitan-Banin et al., 2003) in *cirB*, *cirC*, and *cirD* (JN1, JN2, and JN3, respectively; Table 1) and double knockout strains in *cirB/C*, *cirB/D*, and *cirC/D* (GL1, GL2, and GL3, respectively; Table 1). All strains were verified via PCR (Supplemental Figure 3) and DNA sequencing analysis to ensure the correct knockouts had been created. Despite repeated efforts, a knockout was unable to be generated in *cirA*. Nevertheless, the following results give us a clear picture that each of these genes appears to work together in *H. volcanii* to regulate gene expression in response to light conditions. We performed a comparative transcriptional analysis examining *cir* gene expression in strains JN1, JN2, and JN3 as compared with the *H. volcanii* wild-type parental strain H26. H26 and each of the Δ *cir* strains were cultured as previously described in balanced growth in conditions of LD 12:12. Following this, qRT-PCR was used to examine *cir* gene expression in a Δ *cir* background strain. For example, cDNA prepared from JN1 (Δ *cirB*) RNA was subjected to qRT-PCR using *cirA*, *cirC*, and *cirD* primers. Using this strategy, we were able to ascertain whether gene expression in the three remaining viable *cir* genes was affected in this mutant background. In each knockout strain examined, wild-type *cir* gene expression was arrhythmic in a Δ *cir* background, as compared with wild-type rhythmic expression. An example of arrhythmic gene expression for each mutant strain examined can be see in Figures 4A–C, and can be compared against the rhythmic gene expression demonstrated in wild-type (Figures 3B–D). This pattern of arrhythmic gene expression was seen for every *cir* single knockout examined (data not shown due to number of combinations tested). This consistent pattern of arrhythmic gene expression leads us to speculate that expression of all four *H. volcanii cir* genes are coordinately regulated in response to light/dark conditions. This same loss of rhythmic gene expression was also seen in the double knockout strains GL1, GL2, and GL3 (data not shown). These results are similar to those of previous studies (Ishiura et al., 1998) that reported that loss of any single *kai* gene results in arrhythmicity in *S. elongatus*. Interestingly, growth studies on all six *H*. *volcanii* mutant strains revealed no significant change in growth rate or yield during LD 12:12 conditions (Supplemental Figure 4). Again, these results are reminiscent of work in cyanobacteria reporting that knockouts in any of the three *kai* genes do not affect growth of the organism (Ishiura et al., 1998).

### Restoration of rhythmic gene expression in *cir* knockout strains via complementation

After demonstrating that a single *cir* gene knockout disrupts rhythmic, light-dependent gene expression among the remaining wild-type *cir* genes, we next performed a series of complementation analyses. For this, we introduced, *in trans*, a copy of each wild-type *cir* gene on an autonomously replicating plasmid into its corresponding mutant strain. An example of this analysis can be seen in Figure 4D, whereby the full-length, wild-type *cirB* gene was introduced on plasmid pMM2 into the Δ *cirB* strain JN1. Positive transformants were verified by PCR, cultured in LD 12:12 conditions, and examined for the restoration of rhythmic *cir* gene expression. Using this approach, all three *cir* deletion strains were complemented to wild-type levels of light-dependent, rhythmic gene expression (Figure 4D; complementation of JN2 and JN3 not shown due to space limitation).

## Discussion

Circadian clocks confer on organisms the ability to anticipate and respond to recurrent 24 h environmental cycles. The selection pressures to establish an internal temporal order synchronized to the external environment were present from the evolution of the first life forms on earth (Paranjpe and Sharma, 2005). One of these pressures, mutagenic UV solar radiation, which has given rise to the “escape from light” hypothesis for clock evolution (Pittendrigh, 1993; Rosato and Kyriacou, 2002), was significantly more intense during Earth's early history than today (Karam, 2003). Unicellular cyanobacteria, among the most ancient life forms based on microfossil and biomarker records (Nisbet and Sleep, 2001), are the only prokaryotes in which circadian rhythms have been conclusively demonstrated (Johnson, 2007). Their role in the shift of the Earth's atmosphere from a reductive to an oxidative one is well documented (Nisbet and Sleep, 2001). Thus, as photosynthesizers cyanobacteria must seek out light, yet also devise strategies to ameliorate the DNA-damaging effects of UV radiation. Furthermore, the development in non-heterocystous cyanobacteria of oxygenic photosynthesis and nitrogen fixation, two incompatible biochemical processes owing to the O_2-sensitive_ nitrogenase complex, required the temporal separation of these activities (Huang et al., 1999; Berman-Frank et al., 2001; Church et al., 2005). Indeed, the *kaiC* clock gene seems to be ubiquitous among cyanobacterial species, suggesting that daily temporal control of cellular activity is not restricted to photosynthesis and N_2_ fixation in these organisms (Lorne et al., 2000).

What then about other prokaryotes, and in particular the members of the Archaea, the third domain of life? Dating the origin of the Archaea is currently debated, yet some evidence suggests that archaeal methanogens may have been present by 3.4–2.6 billion years ago (Gribaldo and Brochier-Armanet, 2006). These dates coincide with the evolution of the cyanobacteria and thus suggest that the Archaea were exposed to similar selection pressures (e.g., intense UV radiation). Many halophilic archaea synthesize UV-protective pigments, gas vesicles for vertical migration, and/or flagella, all of which could conceivably be subject to circadian-regulated expression. The unique properties of the Archaea make it interesting to consider what molecular components and mechanisms might comprise a circadian system in these organisms. There is no reason to assume that an archaeal circadian system would necessarily be closely related to the cyanobacterial model, as the Archaea are no more closely related to the Bacteria than either domain is to the Eukarya (Woese, 1994). Indeed, archaeal transcriptional and translational mechanisms more closely resemble those of eukaryotes, while archaeal gene structure is more bacteria-like reviewed in (Allers and Mevarech, 2005). Thus, the components of an archaeal circadian system may resemble something between the bacterial and eukaryotic models. Even among the eukaryotic models studied, plants and mammals for example, there are significant clock differences at the molecular level.

To date, most of what we know about the phototactic response of Archaea comes from studies in the extreme haloarchaeon *Halobacterium* sp. NRC-1 (Schimz and Hildebrand, 1985; Hildebrand and Schimz, 1986; Rudolph and Oesterhelt, 1995; Nutsch et al., 2003; Oprian, 2003). Shortly after the publication of the NRC-1 genome (Ng et al., 2000), an *in silico* analysis was performed to identify a subset of genes involved in light sensing in this organism (DasSarma et al., 2001). Among the genes identified was a single *kaiC* homolog, leading the authors to speculate that circadian rhythms may be a property of some Archaea (DasSarma et al., 2001). An extension of these results came with the publication of a microarray analysis by Whitehead et al., demonstrating the global regulation of diurnal gene expression in the transcriptome of NRC-1 (Whitehead et al., 2009). This study, while broadly focused in nature, is clearly an encouraging first step toward defining what a transcription/translation-based circadian response might look like in haloarchaea. Interestingly, the authors reported no change in gene expression in the single NRC-1 *kaiC* homolog in response to 12-h light/12-h dark (LD 12:12) conditions, nor was it directly addressed why this might be. This report is in direct contrast to results we have obtained demonstrating regulation of gene expression in the four *H. volcanii kaiC* homologs in response to LD 12:12 growth conditions. Finally, we must note that an intriguing study recently reported the presence of a circadian oscillation of peroxiredoxin oxidation in *H. salinarum* NRC-1, providing the first evidence of a posttranslational circadian mechanism that appears to be shared among the three domains of life (Edgar et al., 2012).

In conclusion, we have characterized four cyanobacterial, circadian clock *kaiC* homologs from the model haloarchaeon, *H. volcanii*, and have determined that they are transcriptionally regulated in a diurnal, light-dependent fashion. These results are noteworthy as this is the first attempt to directly characterize the gene expression of the ubiquitous *kaiC* homologs found among archaeal genomes. While we have demonstrated the transcriptional control of these four genes by environmental cues of light and darkness, much remains to be discovered as to their functional roles in *H. volcanii* as well as in other Archaea.

### Conflict of interest statement

The authors declare that the research was conducted in the absence of any commercial or financial relationships that could be construed as a potential conflict of interest.

## Supplementary material

Supplemental Figure 1 **Placement of the four *H. volcanii* cir genes in the context of the genome.** The Lasergene core suite (DNASTAR) was used to visualize the immediate genomic neighborhood of the four *H. volcanii* circadian genes based on data obtained from the NCBI GenBank database.

Supplemental Figure 2 **Growth of wild-type *H. volcanii* at 45°C over a 72 h period in LL (**■**) or DD (**○**).** Values are averages taken from duplicate cultures.

Supplemental Figure 3 **Verification of single (A) and double (B) knockout strains via PCR analysis.** Knockout strains (odd-numbered lanes) were compared against the H26 parental background (even-numbered lane) for confirmation of gene deletion. **(A)** Lane 1, molecular weight marker; lane 2, wild type *cirB* (1.2 kb); lane 3, Δ*cirB* (JNI, 700 bp); lane 4, wild-type *cirC* (746 bp); lane 5, Δ*cirC* (JN2, 500 bp); lane 6, wild-type *cirD* (550 bp); lane 7, Δ*cirD* (JN3, 430 bp). **(B)** lane 1, 100 bp ladder; lane 2, wild-type *cirB* (750 bp) and *cirC* (1.2 kb); lane 3, GL1 harboring a deleted portion of *cirB* (500 bp) and *cirC* (700 bp); lane 4, wild-type *cirB* and *cirC* (323 bp); Lane 5, GL2 harboring a deleted portion of *cirB* and *cirD* (201 bp); Lane 6, wild-type *cirC* and *cirD*; Lane 7, GL3 harboring a deleted portion of *cirC* and *cirD*. DNA sequencing analysis was used to confirm these results (data not shown).

Supplemental Figure 4 **Growth curves of *H. volcanii* parental strain H26 as compared with various cir knockout strains. (A)** Growth of H26 as compared with JN1,JN2, and JN3. **(B)** Growth of H26 as compared with GL1, GL2, and GL3.
