# Supplementary figures and images for: Light-dependent expression of four cryptic archaeal circadian gene homologs
Source: Front Microbiol. 2014 Mar 4;5:79. doi: 10.3389/fmicb.2014.00079 (PMC3941300; doi:10.3389/fmicb.2014.00079)

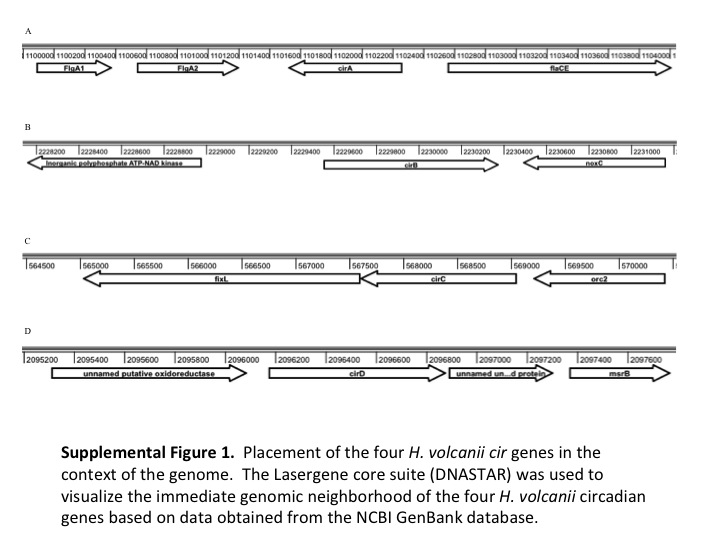

Supplement: Supplemental Figure 1 — Placement of the four H. volcanii cir genes in the context of the genome. The Lasergene core suite (DNASTAR) was used to visualize the immediate genomic neighborhood of the four H. volcanii circadian genes based on data obtained from the NCBI GenBank database. [file Presentation1.ZIP › 79226_Bidle_Suppl_Figure_1.JPEG]

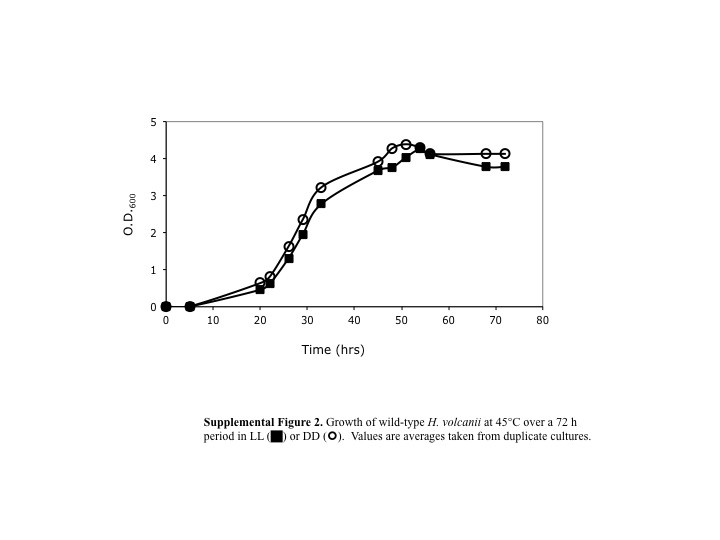

Supplement: Supplemental Figure 1 — Placement of the four H. volcanii cir genes in the context of the genome. The Lasergene core suite (DNASTAR) was used to visualize the immediate genomic neighborhood of the four H. volcanii circadian genes based on data obtained from the NCBI GenBank database. [file Presentation1.ZIP › 79226_Bidle_Suppl_Figure_2.JPEG]

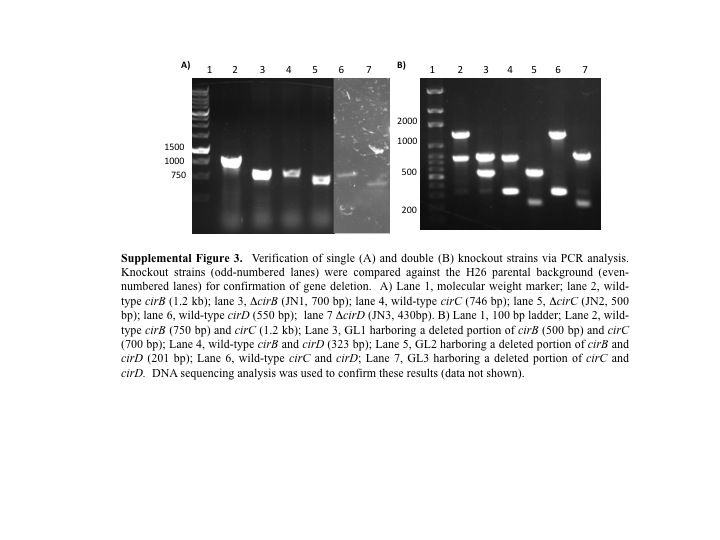

Supplement: Supplemental Figure 1 — Placement of the four H. volcanii cir genes in the context of the genome. The Lasergene core suite (DNASTAR) was used to visualize the immediate genomic neighborhood of the four H. volcanii circadian genes based on data obtained from the NCBI GenBank database. [file Presentation1.ZIP › 79226_Bidle_Suppl_Figure_3.JPEG]

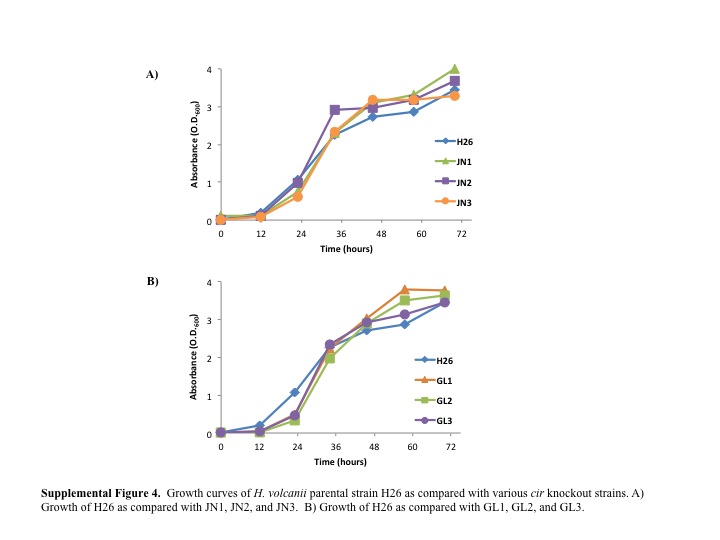

Supplement: Supplemental Figure 1 — Placement of the four H. volcanii cir genes in the context of the genome. The Lasergene core suite (DNASTAR) was used to visualize the immediate genomic neighborhood of the four H. volcanii circadian genes based on data obtained from the NCBI GenBank database. [file Presentation1.ZIP › 79226_Bidle_Suppl_Figure_4.JPEG]
